# Development of Wastewater Pooled Surveillance of Severe Acute Respiratory Syndrome Coronavirus 2 (SARS-CoV-2) from Congregate Living Settings

**DOI:** 10.1128/AEM.00433-21

**Published:** 2021-06-11

**Authors:** Lisa M. Colosi, Katie E. Barry, Shireen M. Kotay, Michael D. Porter, Melinda D. Poulter, Cameron Ratliff, William Simmons, Limor I. Steinberg, D. Derek Wilson, Rena Morse, Paul Zmick, Amy J. Mathers

**Affiliations:** a Department of Engineering Systems & Environment, University of Virginia, Charlottesville, Virginia, USA; b Department of Medicine, Division of Infectious Diseases, University of Virginia Health System, Charlottesville, Virginia, USA; c School of Data Science, University of Virginia, Charlottesville, Virginia, USA; d Clinical Microbiology Laboratory, Department of Pathology, University of Virginia Health System, Charlottesville, Virginia, USA; e Facilities Management, Energy and Utilities Division, University of Virginia, Charlottesville, Virginia, USA; f Facilities Management, University of Virginia Health System, Charlottesville, Virginia, USA; g Health Information & Technology, University of Virginia Health System, Charlottesville, Virginia, USA; University of Bayreuth

**Keywords:** wastewater epidemiology, COVID-19, SARS-CoV-2, pooled surveillance, college dormitory surveillance, congregate living surveillance, wastewater, sewer, sewershed

## Abstract

Wastewater-based monitoring for severe acute respiratory syndrome coronavirus 2 (SARS-CoV-2) at the individual building level could be an efficient, passive means of early detection of new cases in congregate living settings, but this approach has not been validated. Preliminary samples were collected from a hospital and a local municipal wastewater treatment plant. Molecular diagnostic methods were compared side by side to assess feasibility, performance, and sensitivity. Refined sample collection and processing protocols were then used to monitor two occupied dormitory complexes (*n* = 105 and 66) over 8 weeks. Wastewater results were validated using known case counts from external clinical testing of building occupants. Results confirm that ultracentrifugation from a 24-h composite collection had a sensitivity of 96.2% and a specificity of 100%. However, the method could not distinguish new infectious cases from persistent convalescent shedding of SARS-CoV-2 RNA. If the detection of convalescent shedding is considered a false positive, then the sensitivity is 100% and specificity drops to 45%. It was determined that the proposed approach constitutes a highly sensitive wastewater surveillance method for detecting SARS-CoV-2, but it could not distinguish new infectious cases from persistent convalescent shedding. Future work must focus on approaches to distinguish new infections from convalescent shedding to fully realize the potential of building wastewater as a surveillance tool for congregate living.

**IMPORTANCE** Some of the most severe outbreaks of COVID-19 have taken place in places where persons live together, such as nursing homes. Wastewater testing from individual buildings could be used for frequent pooled surveillance of virus from all occupants, including those who are contagious, with or without symptoms. This work provides a sensitive practical method for detecting infected individuals, as validated in two building complexes housing occupants who underwent frequent clinical testing performed by external entities. Although this sensitive method could be deployed now for pooled surveillance as an early warning system to limit outbreaks, the study shows that the approach will require further refinement to differentiate contagious, newly infected individuals from persons who have persistent viral fragments shedding in their stool outside the contagious period.

## INTRODUCTION

Severe acute respiratory syndrome coronavirus 2 (SARS-CoV-2) is a highly contagious respiratory virus with a case fatality rate of 1 to 2% (1). Mask-wearing and social distancing have been shown to reduce the spread of SARS-CoV-2, thereby decreasing incidence of coronavirus disease 2019 (COVID-19), the illness caused by SARS-CoV-2 (2). Because people are unable to wear masks or social distance at all times when they live together, congregate living settings (e.g., nursing homes, prisons, etc.) have been disproportionately impacted by large outbreaks (3–6). Approaches to limit transmission in such settings have included aggressive entry screening via wellness attestations and/or temperature checks (7, 8); however, the effectiveness of these strategies has been hampered by the fact that an infected individual is contagious prior to or without ever exhibiting symptoms (9–11). Frequent clinical testing of all occupants could be an effective means of identifying and separating contagious individuals and quarantining others (3, 4, 12). However, there are significant logistical considerations associated with repeated mass testing, such as cost, personnel availability, and exposure risk, test availability, privacy, compliance, testing fatigue, occupant anxiety, and others (13). To prevent outbreaks in congregate living settings, there is urgent need for frequent passive pooled surveillance to quickly detect new cases in a building. Finding a new positive in a pooled sample could then trigger more targeted additional individual testing with subsequent rapid isolation of infected persons and their close contacts before an outbreak can occur (14, 15).

Widespread detection of appreciable quantities of largely nonviable SARS-CoV-2 RNA in stool, coupled with the strong need for passive routine surveillance, has prompted strong interest in wastewater-based testing (WBT) as means to monitor for virus prevalence (16, 17). Recently published work documented the potential usefulness of this strategy at a community level (e.g., via sampling at a municipal wastewater treatment facility) (18, 19, 21–23). A working group comprising water and wastewater professionals from academia and public practice have asserted that, “wastewater surveillance in sewersheds is a rapidly developing area of research that has the potential to inform public health policy decisions in the context of the current pandemic” (24).

There is also rapidly emerging interest in wastewater-based monitoring for SARS-CoV-2 RNA at the individual building level for congregate living settings (25, 26). There have been popular (lay) press reports about how this approach has been implemented for various university dormitories; e.g., at the University of Arizona, Syracuse University, and Pennsylvania State University. However, there is no agreement on what constitutes best practices for implementation of this approach (27). Among this group, work from Syracuse University offers the greatest amount of technical detail, but additional investigation is still needed (26).

The overarching objective of this work was to evaluate the hypothesis that frequent monitoring of pooled wastewater samples could be an efficient means of monitoring for COVID-19 cases among building occupants and guide strategic allocation of testing resources. Three specific objectives were pursued: (i) establishing a robust strategy for collecting representative wastewater samples, (ii) comparing and refining molecular diagnostic techniques, and (iii) sampling from occupied congregate living settings and validating wastewater results based on external clinical testing. Wastewater samples were collected from several populations of individuals undergoing frequent clinical testing for COVID-19, in a hospital and in several occupied university dormitories. In addition, we assessed the methods on samples from a municipal wastewater treatment facility and the municipal drinking water distribution system.

## RESULTS

### Establishing a robust sampling protocol.

Hospital wastewater was initially collected from manholes as grab samples. However, these samples repeatedly gave definitively negative results (data not shown). Therefore, dye testing was used to investigate hydraulic connectivity between patient rooms and several candidate sampling sites. Two initial dye tests revealed that wastewater from the COVID-19 unit was not accessible via the manhole locations that had been first attempted. This outcome prompted a third round of dye testing at an indoor cleanout valve located just upstream of where the wastewater exits the building. Observed transit time to this location was 2 to 3 min. All subsequent hospital samples were collected from this location.

It was then decided that composite samples would likely be more representative than grab samples. Therefore, autosamplers were used to collect overnight composite samples, typically spanning periods of 22 to 24 h. A typical sampling program was 30 ml collected every 15 min, such that the expected volume for a 24-h composite was ∼2.9 liters. The average daily flow rate for days on which hospital samples were collected was 9.0 to 13.5 gal per min, with recurrent periods of very low flow or no flow, during which autosamplers were unable to collect the specified sample volume. The marked variability in wastewater flow rate made it such that different volumes were collected on different sampling dates. All hospital wastewater samples greater than ∼200 ml were considered usable for preliminary evaluation of molecular analyses, as described below; that is, no overnight samples were excluded because they were significantly greater than or less than the expected volume.

Daytime temperatures during the study period were routinely as high as 38°C (101°F). The autosamplers were portable instruments, in that they did not have wall outlet power adaptors; moreover, most sampled locations were not near a power supply. It was therefore necessary to pack the sample jars on ice during the overnight sample collection intervals. At least a small amount of ice was usually present when the samples were collected on the following day.

### Comparing and refining molecular diagnostic techniques.

Hospital wastewater samples, as well as raw influent and primary solids from the local municipal wastewater treatment plant (WWTP), were used to compare published concentration and extraction protocols for SARS-CoV-2 analysis in wastewater (22, 28–31) (Table 1). An immediate observation from these results is that SARS-CoV-2 was detected in all three categories of positive-control wastewaters, wherein it was expected that SARS-CoV-2 would be present at appreciable concentrations. However, the various methods gave different results.

**TABLE 1:** *C_T_* values for preliminary samples from hospital cleanout valve and the local municipal WWTP, processed using various concentration and extraction methods from literature^*a*^

Source	Extraction	*C_T_* value														
		EP filtration			Ultracentrifugation			PEG precipitation			Biobot PEG			No concn		
		N1	N2	RP	N1	N2	RP	N1	N2	RP	N1	N2	RP	N1	N2	RP
Hospital	None	**31.2**	**32.8**	**33.3**												
	QiaAmp without DTT	**33.4**	**33.2**	**ND**^*b*^	**33.3**	**39.9**	**35.3**	ND	35.2	ND	ND	ND	ND			
	QiaAmp with DTT^*c*^				**36.5**	**34.7**	**32.6**									
	NucleoSpin	**28.9**	**29.5**	**31.0**	**30.9**	**32.5**	**30.1**	**31.4**	**31.3**	ND*	**36.7**	**37.1**	**35.1**			
WWTP raw influent	QiaAmp without DTT				ND	ND	34.4	35.5	ND	ND	ND	ND	ND			
	QiaAmp with DTT^*c*^				38.2	ND	32.9									
	NucleoSpin				**30.3**	**31.2**	**28.3**	**33.7**	**32.5**	**33.3**	ND	ND	ND			
WWTP primary solids	QiaAmp without DTT													36.2	ND	ND
	QiaAmp with DTT^*c*^													34.7	36.2	40.1
	NucleoSpin													**31.8**	**32.5**	**34.6**

a All methods were applied to subsamples of a composite sample collected on a single day in early July 2020 and processed the same day. Boldface indicates positive determinations. Blank cells correspond to combinations of protocols that were not assessed. ND, not detected (*C_T_* ≥ 45).

b Sample inhibition occurred, but the sample was positive for the presence of SARS-CoV-2 RNA.

c Postextraction treatment with DTT prior to qPCR.

Regarding concentration, it was observed that both polyethylene glycol (PEG) precipitation methods gave relatively poor performance in this study. However, both electropositive filtration and ultracentrifugation yielded positive results across the tested hospital and WWTP samples. Choice of RNA extraction method also had a strong impact on detectability of SARS-CoV-2. The NucleoSpin protocol yielded 7 positive results in 8 trials across all three wastewater categories (hospital, WWTP raw influent, and WWTP primary solids). The QiaAmp protocol with and without dithiothreitol (DTT) yielded only 4 positive results in 11 trials (Fisher’s exact test, *P* = 0.04). None of the positives corresponded to raw influent samples. Six of the 11 QiaAmp and 2 of the 8 NucleoSpin (Fisher exact test, *P* = 0.21) samples failed internal quality control (QC), which monitors sample inhibition of the enzymatic PCR assuming all samples would have a positive RNase P (RP) target. Postextraction treatment of the QiaAmp samples with DTT improved detectability; i.e., it decreased cycle threshold (*C_T_*) value, and/or transformed an inhibited/failed sample into a positive result. However, for samples treated using the same concentration protocol, NucleoSpin extraction gave lower *C_T_* values than QiaAmp extraction followed by DTT treatment (Wilcoxon signed-rank test, *P* < 0.0001) (Table 2).

**TABLE 2:** Ultracentrifuge concentration with comparison of two extraction kits from multiple wastewater sources

Source	Sample day	*C_T_* value					
		QiaAmp			NucleoSpin		
		N1	N2	RP	N1	N2	RP
Hospital	Baseline without DTT	33.25	39.85	35.31	30.90	32.54	30.14
	Baseline with DTT	36.46	34.71	32.62			
	8	33.51	34.46	31.27	30.62	33.18	31.01
	9	38.72	39.39	30.10	39.09	37.50	31.37
	9 (duplicate)	35.90	37.45	27.48	38.37	38.56	28.17
	12	ND	ND	32.27	38.35	33.94	31.13

WWTP raw influent	Baseline without DTT	ND	ND	34.42	30.26	31.23	28.32
	Baseline with DTT	38.19	ND	32.94			
	8	53.24	40.58	32.20	34.01	ND	30.26

WWTP primary solids	Baseline without DTT	36.22	ND	ND	31.76	32.46	34.62
	Baseline with DTT	34.75	36.21	40.1			
	8	32.02	36.26	31.36	30.79	32.42	30.85

Dormitory	8	ND	38.85	30.89	34.70	37.99	30.61
	8 (duplicate)	ND	ND	30.89	32.58	35.58	31.29
	14	39.06	ND	32.93	37.30	39.32	30.38
	14, cleanout valve	ND	ND	33.56	ND	ND	32.51
	14, cleanout valve (duplicate)	ND	ND	36.87	ND	ND	34.78

Based on results from the side-by-side method comparison and other logistical factors (most notably sample volume; see Discussion), concentration via ultracentrifugation followed by NucleoSpin extraction was selected for continued use in this study. For all subsequent sampling, overnight composite samples were fully homogenized (mixed) inside the sample collection jar; then, 50-ml volumes were transferred to sterile tubes and transported on ice to the laboratory. The rest of the wastewater was poured back into the manhole or cleanout valve where it came from, to avoid transporting and later disposing of large quantities of biological waste.

Additional comparison of ultracentrifugation followed by each of the evaluated RNA extraction kits demonstrated that the NucleoSpin kit generally had lower *C_T_* values of the RP internal control and less PCR sample inhibition for wastewaters from hospital, WWTP, and occupied dorms than the QiaAmp extraction (Table 2).

### Sample collection from occupied buildings and validation/interpretation of results.

Additional samples were collected from hospital and WWTP using the finalized sample collection and protocols described above. A sample of municipal drinking water from the same distribution network was also processed using the same protocols. Table 3 summarizes *C_T_* values for these samples alongside relevant case counts. Wastewater samples were also collected from two occupied dormitory complexes using these protocols. Table 4 summarizes *C_T_* values for these samples alongside relevant case counts. As the study was blind to the specifics of clinical testing, there was no intentional temporal alignment of dorm occupants and wastewater testing. The interval between successive clinical and wastewater-based testing was never more than 8 days. Positive wastewater results were obtained 8 days after the first positive clinical results and 5 days after the second set of clinical positive results.

**TABLE 3:** Sampling results for hospital and the local municipal wastewater treatment plant over time

Source	Sample day^*a*^	*C_T_* values (N1, N2, RP)^*b*^	Determination	Anticipated result based on occupancy or epidemiology	Interpretation	No. of cases^*c*^
Hospital	Baseline^*c*^	30.9, 32.5, 30.1	Positive	Positive	True positive	30
	8	30.6, 33.2, 31.0	Positive	Positive	True positive	28
	9	39.1, 37.5, 31.4	Positive	Positive	True positive	28
	9 (duplicate)	38.4, 38.6, 28.2	Positive	Positive	True positive	
	12	38.3, 38.9, 31.2	Positive	Positive	True positive	33
	35	32.2, 33.5, 32.2	Positive	Positive	True positive	34
	35 (duplicate)	32.7, 34.1, 34.2	Positive	Positive	True positive	
	50, subsample a^*e*^	34.8, 37.6, 30.4	Positive	Positive	True positive	30
	50, subsample b^*e*^	41.9, 41.2, 33.4	Positive	Positive	True positive	
	51	38.9, 41.0, 28.3	Positive	Positive	True positive	29
	52	37.7, 40.3, 36.3	Positive	Positive	True positive	29

WWTP, raw influent	Baseline^*d*^	30.3, 31.2, 28.3	Positive	Positive	True positive	7.9
	8	34.0, ND, 30.3	Indeterminate	Positive		23.3
	21	34.2, 35.2, 34.5	Positive	Positive	True positive	22.4

WWTP, primary solids	Baseline^*d*^	31.8, 32.5, 34.6	Positive	Positive	True positive	7.9
	8	30.8, 32.4, 30.9	Positive	Positive	True positive	23.3
	21	36.2, 39.2, 35.0	Positive	Positive	True positive	22.4
Tap water	14	ND, ND, ND	Negative (no human DNA anticipated)	Negative	True negative	NA

a End of overnight sample collection period, which is also the same day samples were processed. Numbering of days was from the start of the study period in early July.

b ND, not detected (*C_T_* ≥ 45).

c Number of COVID-19 patients in the hospital tower during sample collection (hospital samples); 7-day moving average of new cases for WWTP catchment area (city of Charlottesville) (https://globalepidemics.org/key-metrics-for-covid-suppression/). NA, not applicable.

d From Table 1.

e The overnight composite sample was divided. Subsample a was processed the day it was collected. Subsample b was refrigerated and processed 24 h later.

**TABLE 4:** Sampling results for two UVA dorm complexes over time

Complex	Sample day^*a*^	*C_T_* values (N1, N2, RP)^*b*^	Determination	Interpretation^*c*^	No. of wkly cases^*d*^
A	8	34.7, 38.0, 30.6	Positive	True positive	103 N, 2 NP [both day 8 after positive], 2 I, 0 C
	8 (duplicate)	32.6, 35.6, 31.3	Positive	True positive	

	14	37.3, 39.3, 30.4	Positive	True positive	102 N, 1 NP [day 5 after new clinical positive], 1 I, 2 C

	14, cleanout valve R	ND, ND, 32.5	Negative	True negative	∼20 N, 0 NP, 0 I, 0 C
	14, cleanout valve R (duplicate)	ND, ND, 34.8	Negative	True negative	

	21	33.4, 35.3, 35.3	Positive	Positive, conv. only	102 N, 0 NP, 0 I, 3 C
	23, cleanout valve A	ND, ND, 31.9	Negative	Positive, conv. only	∼15 N, 0 NP, 0 I, 1 C (WW tested 16 days after single clinical positive)
	31 (same day)	35.3, 35.7, 30.3	Positive	Positive, conv. only	102 N, 0 NP, 0 I, 3C
	31 (next day)	ND, 36.9, 31.3	Indeterminate		

	32	ND, 36.5, 29.1	Indeterminate		
	32 (duplicate)	36.2, 37.6, 29.8	Positive	Positive, conv. only	

	36	38.1, 38.9, 33.4	Positive	Positive, conv. only	102 N, 0 NP, 0 I, 3C
	36 (duplicate)	37.3, 40.5, 33.2	Positive	Positive, conv. only	

	42	34.7, 35.1, 30.5	Positive	Positive, conv. only	102 N, 0 NP, 0 I, 3C
	42 (duplicate)	35.5, 36.4, 31.2	Positive	Positive, conv. only	

	45	34.4, 35.2, 31.1	Positive	Positive, conv. only	
	45 (duplicate)	35.4, 35.8, 31.1	Positive	Positive, conv. only	

	50	37.6, 41.5, 33.1	Positive	Positive, conv. only	102 N, 0 NP, 0 I, 3C
	50 (duplicate)	37.2, 39.5, 35.4	Positive	Positive, conv. only	

	51	36.0, 37.4, 30.5	Positive	Unknown	Unknown^*e*^
	52	ND, ND, 37.1	Negative	Unknown	Unknown^*e*^
	57	ND, 40.5, 32.5	Indeterminate	Unknown	Unknown^*e*^
B	37	ND, ND, 32.2	Negative	True negative	66 N, 0 NP, 0 I, 0 C
	37 (duplicate)	ND, ND, 31.2	Negative	True negative	

	38	ND, ND, 32.1	Negative	True negative	66 N, 0 NP, 0 I, 0 C
	38 (duplicate)	ND, ND, 32.0	Negative	True negative	

	45	ND, ND, 30.9	Negative	True negative	66 N, 0 NP, 0 I, 0 C
	45 (duplicate)	ND, ND, 31.2	Negative	True negative	

	57	ND, ND, 30.1	Negative	Unknown	Unknown^*e*^

a End of the overnight sample collection period, which was also the day samples were processed. Numbering refers to time since the start of the study, aligning with Table 3. Unless otherwise noted, samples were collected from the manhole locations identified in Fig. 1.

b Lower *C_T_* values indicate higher detected concentrations. ND, not detected (*C_T_* ≥ 45).

c conv., convalescent.

d N, negative; NP, newly positive case identified via positive clinical result (clinical testing dates are in brackets); I, infectious cases corresponding to individuals within 1 to 14 days of their positive clinical result (including NP cases); C, convalescent cases, corresponding to occupants who were >15 days beyond a positive test result. All positive individuals self-isolated within the catchment area for the duration of their illness.

e New- and convalescent-case counts are unknown for the occupant changeover period.

An overarching observation from Tables 3 and 4 is that wastewater results are highly consistent with known presence or absence of COVID-19 cases detected via clinical testing. Across all hospital and dorm samples collected after completion of the side-by-side method comparison, outcomes were as follows: 25 true positives, 0 false positives, 9 true negatives, and 1 apparent false negative. Corresponding sensitivity was 96.2% and specificity was 100% (94.74% and 100%, respectively, if duplicate samples are excluded). These determinations are based on assessment that all positive results from the manhole sampling site in complex A are true positives, even though some known positives within the dormitory sampling were beyond the CDC’s recommended 10-day isolation period as of day 31 of this study (32). All three positive cases occurring in complex A during the study interval were asymptomatic.

If detection of cases that are no longer considered actively contagious is construed as false positives, revised outcomes are as follows: 13 true positives, 12 false positives, 10 true negatives, and 0 false negatives. Then, method sensitivity is 100% and specificity drops to 45%. These results highlight the significance of persistent convalescent shedding, as the method cannot distinguish individuals who are currently symptomatic and infectious from those who are asymptomatic and recovered and continue to shed detectable virus into their stool for many weeks (32).

Regardless, it was observed that wastewater results from both dorm complexes transitioned back to negative after the summertime occupants moved out and new occupants moved in. Incoming occupants were required to undergo clinical testing before or immediately upon moving in, such that no new positive cases should have been present at the start of the fall semester, which was also the last week of this study. This presumption is consistent with negative wastewater results from both complexes on days 51 to 57. It is notable that residual, detectable SARS-CoV-2 did not remain in the wastewater conveyance system once the individual(s) shedding the virus was no longer present in the catchment.

Tables 3 and 4 also offer nuanced results pertaining to: the effect of building population size on sample collection protocol, reproducibility of sample processing methods, influence of sample processing time, and lack of discernible correlation between measured *C_T_* values and known new case counts. These observations are outlined below.

First, results from secondary sampling locations within complex A (i.e., cleanout valves A and R) illustrate that is it possible to monitor multiple locations within a single building at the same time but that measurements become unreliable under very-low-flow conditions (Fig. 1). In a test designed to probe what minimum number of occupants is required to deliver reliable results, a sample collected from cleanout valve A (day 23) yielded a negative result, whereby the wastewater-based determination was negative, even though a known previously positive individual was present on the sampled date (the positive test date was 16 days before the wastewater test date, so the individual may have just resolved shedding). The sampled population size from that cleanout value was 15 persons that day. The corresponding flow rate was very low (rarely covering the sampling holes on the probe), which made it difficult to collect any appreciable sample volume. In a separate test, a sample collected from cleanout valve R (day 14) also gave a negative result, and no known or previously positive persons were present in that building zone. Although the sampled population sizes in both tests were nearly the same, the flow rate in cleanout valve R was much higher and more consistent than in cleanout valve A, which made it possible to collect a representative composite sample.

**FIG 1:**
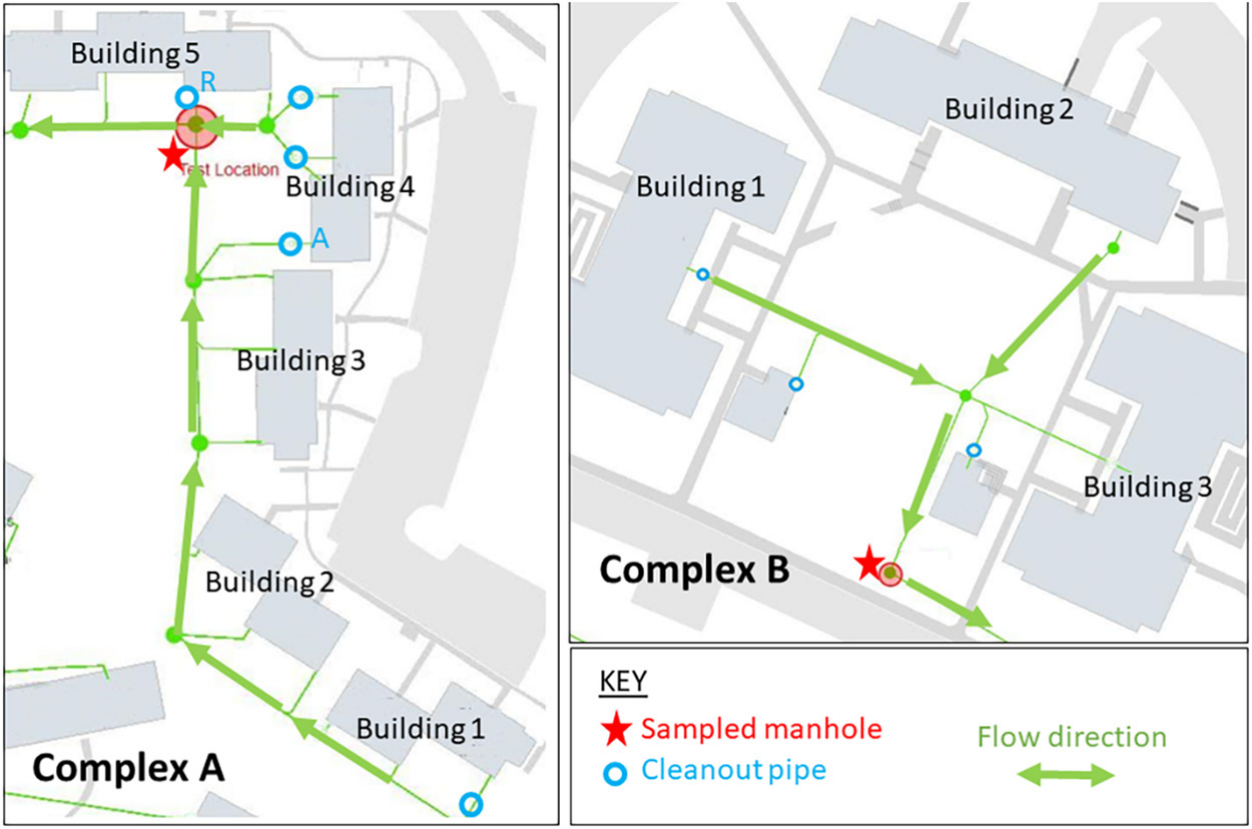
Maps of dormitories in complex A (left) and complex B (right). Red stars denote each sampled manhole. Arrows indicate flow directions. Cleanout valves A and R are secondary testing locations (via cleanout pipes) for selected buildings in complex A.

Reproducibility was evaluated by testing duplicate subaliquots of single samples collected from the hospital and dorms. Two sets of duplicates were collected from the hospital (days 9 and 5), seven sets were collected from dorm complex A (days 8, 14, 32, 36, 42, 45, and 50), and three sets were collected from dorm complex B (days 37, 38, and 45). Of these 12 pairs, all but one yielded consistent determinations (i.e., both positive or both negative). The only exception was from complex A (day 32), whereby one sample was positive, but the other was indeterminate. Of the 11 cases in which duplicates agreed with each other, measured *C_T_* values were within ±1.5 for the pair, which indicates good reproducibility.

The influence of sample processing time was evaluated for two samples, one from the hospital (day 50) and one from dorm complex A (day 31). The hospital sample showed an increased *C_T_* value (indicative of decreasing viral RNA concentration) when it was processed the next day versus the same day. The dorm sample exhibited positive results when it was analyzed on the same day but transitioned to indeterminate when it was analyzed the next day.

Finally, *C_T_* measurements for positive wastewater samples were compared with corresponding COVID-19 case counts (Tables 3 and 4). There was a very weak negative correlation (*R* = −0.2, *n* = 7) between *C_T_* values and known hospital case counts by date for both the N1 and N2 amplicons. There was no apparent correlation when either or both sets of WWTP samples were also considered. *C_T_* values for the N1 and N2 amplicons were highly correlated with each other (*r* ∼ 0.94, *n* = 19) across each kind of wastewater sample (hospital, raw influent, and primary solids) and for all samples combined.

## DISCUSSION

The motivation for this study was to evaluate the usefulness of pooled-wastewater-based testing for COVID-19 surveillance in occupied congregate living settings. Although the number of samples collected was relatively small, the results offer practical lessons about how to collect representative samples and generate usable results. The results also shed light on additional technical and logistical considerations that must be addressed.

### Establishing a robust sampling protocol.

Several key outcomes from this study pertain to practical considerations associated with collection of representative wastewater samples. First, dye testing was an important means of confirming that wastewater from a particular building or wing flows to a candidate testing site. Observed transit times were also much shorter than anticipated by University of Virginia (UVA) facilities management personnel based on building drawings. These unexpected outcomes illustrate the value of preliminary hydraulic testing to confirm sampling location and characterize building flow characteristics before routine surveillance begins.

Second, this study quickly pivoted from grab samples to overnight composite samples. This decision was made based on consultation with representatives from the wastewater industry (e.g., Rivanna Water and Sewer Authority [RWSA] personnel), reference to emerging literature on wastewater-based epidemiology (22, 33), and the presumption that building occupants would produce stools at different times and viral RNA would be transient in the system. It was hypothesized that collecting small volumes of wastewater throughout the day would increase the likelihood of intercepting fecal material from as many building occupants as possible. The results from this work cannot be used to conclude that grab samples will not work for wastewater-based testing; however, flow measurements from the hospital illustrated clear daily trends in wastewater generation by building occupants. Visual observations throughout the day at the sampled dorm locations revealed similar trends; e.g., highest flow volumes and largest quantities of toilet paper debris and presumed fecal matter were evident first thing in the morning. For this reason, care was taken to make sure that this period was always represented in the collected composite samples (33).

A related possible shortcoming of the proposed wastewater-based testing approach is that not all individuals produce a stool every day and/or building occupants may use bathroom facilities in another building. This shortcoming is potentially mitigated by very frequent testing (i.e., if a positive case is missed on one day, it might be caught the next day). Another shortcoming may be that not all infected individuals shed virus in their stool. A small number of published studies with small to moderate sample sizes reveals that only about half of hospitalized patients have detectable SARS-CoV-2 in their stool. Because these studies were based on hospitalized patients, it is still unclear what fraction of asymptomatic cases shed viral fragments via feces (34). More information on SARS-CoV-2 prevalence in stools from symptomatic and asymptomatic cases and what concentrations are shed in stools over time during the progression of their infection would be valuable. Ultimately, if infected individuals do not produce a stool within the building during the sampled interval, or if they are not shedding detectable virus in their stool, this method will not be as effective as desired, but we did not find this to be an issue in this small study.

Another outcome of interest related to sample collection pertains to the influence of population size on virus detectability. The hospital and both dorm complexes offered multiple sampling locations, such that it was possible to monitor different population sizes within the same building. This flexibility was useful for evaluating how big or small the sampled population size could be to detect a known positive case. If the expected COVID-19 prevalence among building occupants is moderate to high, it is valuable to collect samples from relatively small groups (e.g., a single small building or several zones concurrently using multiple cleanout pipes), such that it is not necessary to conduct hundreds of clinical tests to find and isolate the infected individual(s) once a positive result has been obtained. On the other hand, if there is low expected prevalence, it may be more efficient to monitor larger populations (e.g., one large building or two or more connected buildings). The apparent false-negative result obtained from cleanout valve A in complex A illustrates that there may be a practical lower limit to the population size that can be screened using this approach. This limit is not a fixed value; rather, it corresponds to whatever number of persons yields consistent, appreciable wastewater flow, such that a representative composite sample can be obtained. It has been estimated that wastewater-based testing at the community level (i.e., from centralized WWTPs instead of from individual buildings) may be able to detect COVID-19 prevalence as low as 1 positive case per 10,000 persons (18).

The results recorded in this study were binary (positive/negative), with no discernible quantitative correlation between measured *C_T_* value and known new case counts in the corresponding sample catchment. This lack of correlation likely reflects multiple factors, several of which are related to sample collection protocols. First, the aforementioned variabilities in stool generation by person per day, viral shedding into stool by case and over time during a single case, wastewater flow rate over time, and collected volume per composited duration make it such that widely different quantities of virus could be present in a daily composite sample from a single building on the same day. As noted above, significant variability in sample volume was observed. Because this likely corresponded to variability in virus concentration, it is difficult to correlate SARS-CoV-2 concentration and number of new cases. Second, wastewater contains many constituents, such as cleansers and disinfectants from varied housekeeping practices, that could contribute to viral RNA degradation over time (22, 24). It is unknown to what extent these agents could influence detectable SARS-CoV-2 quantity.

Finally, packing samples on ice during collection and transport seems to have been adequate for this study, inasmuch as accurate positive results were obtained for all dorm samples collected from the primary sampling location. Still, it was observed for two samples (one from a hospital and one from a dorm) that SARS-CoV-2 RNA detectability within a raw wastewater sample decreases over time. Analyzing the possible influence of sample processing time was not a key focus for this study, but it is of interest to understand how it and related logistical factors (e.g., sampling handling, storage temperatures, etc.) affect the sensitivity of this approach. This information is especially relevant for assessing the feasibility of testing strategies that rely on shipping wastewater samples to a centralized facility offering fee-based testing services (22, 35).

### Comparing and refining molecular diagnostic techniques.

This study also yielded several outcomes related to sample processing methodology. First, the tested concentration methods yielded variable results. This observation was anticipated based on previous work (31, 33, 36). However, it was somewhat unexpected that ultracentrifugation with a sucrose cushion (26) was about as effective as electropositive filtration, since the former method had not been as widely documented in relevant literature (36). Because the performances of these two methods were equivalent the decision to move forward with ultracentrifugation came down to practical considerations. Regarding benefits, the method is highly sensitive and does not require specialized reagents or costly, scarce consumables. It also makes use of small sample volumes, which is convenient for transport and reduces the amount of hazardous waste that must be disposed of afterwards. A critical drawback is that ultracentrifuges are very costly. Also, because centrifugation is a batch process (i.e., only a fixed number of samples can be spun at the same time) and long spin-up and spin-down times are required, this approach is only moderately scalable. As a result, individual labs may struggle to process the large numbers of samples arising from testing multiple buildings multiple times per week.

It was also observed that choice of RNA extraction protocol is critical for obtaining useful results. Again, this outcome was not surprising based on relevant literature (22, 27, 33, 37). Although only two commercial kits were compared, it was observed that NucleoSpin worked better than QiaAmp, and some samples were still inhibited. Though results are not presented in detail, samples from this study were run together with clinical specimens from UVA hospital (and elsewhere), on several PCR platforms, including multiple commercial platforms, such as Abbott Alinity and m2000. It was observed that samples routinely failed on commercial platforms with built-in extraction protocols. These failures were attributed to PCR inhibition by unknown wastewater constituents (22).

The CDC protocol for SARS-CoV-2 analysis was not originally intended for wastewater-based testing (38). The presence of detectable SARS-CoV-2 RNA in a wastewater sample does not necessarily mean that it contains viable virus that is capable of transmitting COVID-19 (39). However, it was convenient for UVA to process its clinical and wastewater samples together, subject to the same quality control procedures. Clinical diagnostics require very high sensitivity, which is also valuable in the context of pooled-wastewater-based surveillance, in order to get ahead of an outbreak. Additional efforts to improve recovery and make the method more quantitative (e.g., estimate minimum level of detection for the tested combinations of concentration and extraction protocols) would be valuable in future work. A recent review by Ahmed et al. (33) highlighted the urgent need for standardization of process controls during concentration and extraction and assessing extent of inhibition during PCR. The authors noted that most existing studies of wastewater-based SARS-CoV-2 surveillance did not provide any information on recovery efficiency and that there was widespread variability in documented approaches. They also noted that it would be helpful for future related work to include internal process controls, making use of a virus that is morphologically and genetically similar to SARS-CoV-2, relatively safe for lab use, and not normally present in sanitary wastewater, e.g., murine hepatitis virus, bovine coronavirus, feline infectious peritonitis virus, or others.

### Validation/interpretation of wastewater-based testing results.

The results from this study reveal that wastewater-based testing was able to accurately determine when a small number of asymptomatic cases were present. This is notable because existing literature on wastewater-based epidemiology for COVID-19 had not previously documented that it was possible to detect asymptomatic cases (22, 33). Moreover, there is preliminary evidence that SARS-CoV-2 shedding into stool may be more widespread among milder cases and/or younger patients (40). If this observation is borne out in additional work, it could make wastewater-based testing particularly appealing for use in college dormitories.

However, poor correlation between measured *C_T_* values and known new case counts was observed for hospital, WWTP, and occupied-dorm samples collected during this study. This outcome was somewhat unexpected, but it makes sense after the fact based on results from this study and emerging literature. First, for reasons described above, variability during sample collection gives rise to changes in virus quantity that are not related to new-case count. Also, the methods used in this study were not quantitatively calibrated. Finally, viral shedding into stool is known to vary widely among infected individuals and over time in a single individual. It is estimated that 30 to 75% of individuals with COVID-19 shed detectable virus into their stool (10^2^ to 10^8^ gene copies per ml), with or without concurrent gastrointestinal symptoms, during symptomatic, asymptomatic, and presymptomatic cases (22, 39). There is wide variability in the interval between virus detectability in respiratory samples and that in stool samples. In some instances, virus has been first detected in both samples on the same day (41). Much more frequently, there is delay of at least 1 to 3 days, sometimes extending to more than a week, before virus is detectable in stool (17, 41, 42).

It has been well documented in the literature that viral shedding in stool can extend weeks beyond the 10-day infectious period and/or negative respiratory samples. The timing of the positive cases in complex A, where no new cases were recorded after the first 2 weeks of wastewater testing, offers clear indication that persistent convalescent shedding complicates interpretation of wastewater testing results. The last new positive case was identified via clinical testing on day 9, which means that the recommended isolation period for all infected occupants would have ended no later than day 19. However, positive wastewater testing results were obtained until the summertime occupants moved out of the complex. The positive results obtained after day 21 are attributed to persistent convalescent viral shedding from a relatively small number of occupants (3 of 105). There was no apparent trend in *C_T_* values over time after discovery of the last new case. That is, there was no indication that *C_T_* values gradually increased as the infected individuals convalesced and likely decreased their virus shedding. This would have made it challenging to detect a new case via wastewater-based testing during the latter part of the study period, since there would be no way to distinguish between convalescent and new infectious cases. However, the rapid transition back to negative results, as measured within several days after move-out of the summertime occupants, indicates that detectable virus does not persist for long periods in the sewage collection system.

The goal for this work was to evaluate whether wastewater-based testing could be a useful early-warning tool to mitigate COVID-19 outbreaks in congregate living settings by helping decision-makers strategically decide when they should deploy point prevalence testing (i.e., testing all building occupants to find and isolate the infected individual[s]). Ultimately, there are other testing modalities for individuals but they all require individual active collection and specimen and result management. The advantage of wastewater-based testing is one of passive surveillance to signal the need to individual testing when SARS-CoV-2 RNA is detected in the wastewater.

In conclusion, results from this pilot study confirm that it is feasible to deploy wastewater-based testing at the individual building level as a means of implementing passive pooled SARS-CoV-2 surveillance for occupied congregate living settings. The molecular methods used in this study, based on the literature and existing clinical methodology, are highly sensitive, but they are not specific for new infections. Persistent convalescent shedding constitutes an important technical challenge that must be overcome in future work. Possible strategies could include refinement of the molecular methods to make them more robustly quantitative and/or application of statistical modeling approaches based on very frequent testing (i.e., daily samples) to facilitate simulation-based differentiation between new and convalescent cases. Improved interpretation of the results will increase the likelihood that wastewater-based testing will be useful as an early warning system in university dormitories, skilled nursing facilities, and prisons.

## MATERIALS AND METHODS

### Sampled settings.

**(i) Hospital.** The hospital setting was a newly constructed tower at University of Virginia (UVA) Medical Center. This tower contains 84 rooms over three occupied floors but was at only roughly 40% occupancy during this study. During the study period, it was used for isolation and/or treatment of only patients in isolation or quarantine for COVID-19. All rooms are single occupancy, with a separate attached bathroom, including toilet and sink. Mobile patients used the toilets. Stools from immobile patients were collected via rectal tube, bedpan, or commode and disposed of via the toilet. However, small stools were sometimes captured on pads that were disposed of into the solid waste stream. Dye-based point source tracking was performed to confirm that candidate wastewater sampling locations were hydraulically connected to the COVID-19 unit and to estimate wastewater transit time. Approximately 100 ml of a commercial liquid dye (FLT/Yellow-Green; Bright Dyes, Miamisburg, OH) was introduced via a patient room toilet or solid waste hopper. The toilet was then flushed continuously for 2.5 min to promote rapid transport to candidate sampling sites. Visual monitoring at downstream locations was used to confirm flow connectivity and estimate transit times.

**(ii) Wastewater treatment plant.** The Moores Creek Advanced Water Resource Recovery Facility in Charlottesville, VA, is a municipal wastewater treatment plant (WWTP) operated by the Rivanna Water and Sewer Authority (RWSA). Its design flow rate is approximately 15 million gal per day (MGD). Its service population is approximately 143,000. It serves the city of Charlottesville plus some portions of the surrounding Albemarle County.

**(iii) Private residence.** Municipal tap water was collected from a private residence (four full-time occupants) located within the distribution network that serves the UVA hospital and sampled dormitories.

**(iv) Occupied dormitories.** The college dorm settings were two complexes located approximately 0.5 km apart. Both complexes contain multiple apartment-style buildings (Fig. 1). Complex A comprises five individual buildings, each containing 20 units. Each unit contains two single-occupancy bedrooms, one and a half bathrooms, and a shared kitchen. Complex B comprises three individual buildings, each containing 15 units. Each unit contains four single-occupancy bedrooms, a single shared bathroom, and a single shared kitchen. Neither location was at full occupancy during the study interval.

Figure 1, left panel, shows the locations of three sampling sites within complex A: one manhole collecting flow from all five buildings (red star), plus two building-level cleanout pipes (labeled “A” and “R”). Figure 1, right panel, shows the location of the single sampling site in complex B, which was a manhole collecting flow from all three buildings in the complex. Approximately 105 occupants were living in the portions of complex A constituting the catchment for the manhole that was used as primary sampling location for this complex. Sixty-six occupants were living in complex B. Building occupants in both complexes for nearly all of the study period were student athletes. They were required to wear masks and encouraged to practice social distancing and good hand hygiene. Enhanced cleaning protocols were used in the dormitories to avoid widespread transmission.

### Sampling protocols.

**(i) Hospital and occupied dormitories.** Wastewater samples were collected from the aforementioned hospital and dorm locations from 7 July through 2 September 2020. Locations and CDC protocols pertaining to safe handling of sanitary wastewater were observed during all sampling events, with the following modifications (43, 44). All operators wore cloth or paper non-N95 masks, protective eyewear or a face shield, a disposable liquid impermeable gown, and gloves. Alcohol-based hand hygiene was practiced before and after sampling. All equipment surfaces that came into direct contact with wastewater were sanitized using a 10% (vol/vol) bleach solution for at least 5 min of contact time, followed by multiple rinses with tap water. Care was taken to have one person principally access wastewater samples and contaminated equipment while wearing full personal protective equipment (PPE), assisted by another person who did not have direct contact with the waste. The earliest samples from the UVA hospital constituted grab samples, whereby a container (2.5-gal plastic bucket) was attached to a long pole and held underneath the outfall to collect total volumes of 6 to 10 liters, often requiring multiple sequential captures to get adequate volume.

Subsequent samples from hospital and dorms were collected using commercial, off-the-shelf autosamplers (Sigma 900 Max and AS950) (Hach, Loveland, CO). These instruments were programmed to collect small volumes at prespecified timed intervals over periods of approximately 1 day (20 to 24 h). Several different sampling programs were attempted. The interval between successive samples was 10 to 30 min. Collected volumes were 30 to 50 ml per sampling event. For the majority of the study, the standard program was 30-ml samples collected every 15 min. Collection containers were 5-gal glass jars with Teflon-lined screw tops. The autosamplers exhibited highly variability in volume precision and reproducibility, especially at very small draw volumes. This was a known limitation of the instruments, based on the user’s manual. The autosamplers also struggled to collect the specified sample volumes under low flow. Taping over some holes on the sample collection probe (strainer) to exclude air was helpful for improving sample flow; however, there was still noticeable variability in collected volumes. Sample jars were packed with ice during collection. At the end of each sampling interval, samples were immediately transported to a laboratory and processed the same day. Only a single composite sample was collected on each sampling date from hospital or dorm locations, due to the long duration of each sampling interval and the limited availability of autosamplers.

**(ii) WWTP.** RWSA personnel provided 1-liter samples of their composited raw influent as well as 50-ml grab samples of the solids from the underdrain of their primary clarifiers (“primary solids”). Collection of their composited raw influent started at 12 a.m., and samples were collected at roughly 10 a.m. All samples were transported to lab on ice and processed on the same day. Raw influent and primary solids samples were collected from WWTP on three separate days in July 2020.

**(iii) Municipal tap water.** Approximately 60 ml of municipal tap water from a private residence was collected once or twice per hour over a 24-h period. The sample was refrigerated during collection, transported on ice to the laboratory, and processed the same day. Only a single sample of municipal tap water was assessed during this study.

### Molecular methods.

Four wastewater concentration methods were compared for hospital wastewater and WWTP raw influent: (i) electropositive filtration (45), (ii) ultracentrifugation with a sucrose gradient (26), (iii) polyethylene glycol 8000 (PEG 8000) precipitation (46), and (iv) an alternative PEG precipitation based on a protocol from Biobot Analytics (18). No attempt was made to standardize testing volume during preliminary testing; that is, each method was evaluated using whatever volume was reported in the study from which it was taken, but the same composite mixed sample was use to run all comparison assays. No concentration method was applied for the WWTP primary solids, because these samples exhibited rapid self-settling.

For the electropositive filtration method, 6 liters of mixed raw wastewater was passed through a ViroCap filter (Scientific Methods, Granger, IN, USA). Viral material was then eluted in 200 ml of a solution of 1.5% beef extract and 0.05 M glycine (pH 9.5). The supernatant (150 ml) was transferred to a new centrifuge tube, with 1.5 ml of preflocculated skim milk solution. The sample was then stirred for 8 h and then sedimented by centrifugation at 8,000 × *g* for 30 min at 4°C. The supernatant was removed, and the pellet was dissolved in 500 μl of phosphate buffer. This solution then underwent a secondary concentration step via flocculation in a 5% skim milk solution (27, 47).

For the ultracentrifugation method, 40 ml of a mixed raw wastewater sample was added to an ultracentrifuge test tube (Beckman Coulter, Indianapolis, IN). Then, 24 ml of a concentrated sucrose solution (50% sucrose in TNE buffer [i.e., 50 mM Tris-HCl {pH 7.4}, 100 mM NaCl, 0.1 mM EDTA]) was carefully pipetted underneath the sample to create two distinct layers. Samples were spun down in a Beckman Coulter LE80 ultracentrifuge for 45 min at 42,000 rpm (∼150,000 × *g*). Supernatant was decanted, and pellet was resuspended in 300 μl phosphate-buffered saline (PBS) (26).

The third and fourth concentration methods made use of PEG 8000 to precipitate viral material. To apply the method of Hjelmsø et al. (46), 200 ml of mixed raw wastewater was combined with 25 ml of glycine buffer (3% beef extract, 0.05 M glycine; pH 9.6). The mixture was then centrifuged at 8,000 × *g* for 30 min, and the supernatant was filtered using a 0.45-μm polyethersulfone (PES) membrane. PEG 8000 (80 g/liter) and NaCl (17.5 g/liter) were added to the filtered supernatant and incubated with the mixture overnight at 4°C under agitation. Samples were then centrifuged at 13,000 × *g* for 90 min. The resulting pellet was resuspended in 1 ml PBS. For the Biobot method, 120 ml mixed raw wastewater was filtered through a 0.22-μm filter. Filtrate was combined with 12 g of PEG 8000 and 2.7 g of NaCl, shaken for 15 min to fully dissolve PEG/NaCl, and centrifuged at 12,000 × *g* for 1 h. Supernatant was discarded, and the sample was recentrifuged at 12,000 × *g* for 5 min. The resulting supernatant was decanted from the tube, and the pellet was resuspended in 1.5 ml of TRIzol.

Following the concentration step, pellets from all methods were processed using two commercial RNA extraction kits: the QiaAmp viral RNA minikit (Qiagen, Hilden, Germany) and the NucleoSpin RNA Plus kit (TaKaRa Bio USA Inc., USA). The initial volume of the suspended pellets was 0.5 to 1 ml for QiaAmp and NucleoSpin. Again, WWTP primary solids did not require concentration, so both extraction kits were applied directly to the settled solids. Although the quantity of RNA in each pellet was unknown, paired subsamples of the same pellet were processed using both kits, to facilitate meaningful comparison between the two. The final elution volume for both methods was 50 μl. Extracted RNA samples were not assessed for the presence of residual inhibitors.

A subset of samples for the QIAamp kit were processed using dithiothreitol (DTT) as a mucolytic pretreatment step in an attempt to increase RNA recovery. One Thermo Scientific Pierce DTT microtube was rehydrated with 100 μl of nuclease-free water to a final concentration of 500 mM and mixed well. The entire volume was then added to 5 ml cold sterile 0.01 M PBS (pH 7.2), mixed, and used immediately. An equal volume of this solution was then added to the wastewater sample and vortexed. The mixture was incubated at room with intermittent vortexing until liquefied (∼30 min), then eluted in 100 μl, and extracted using the QiaAmp kit according to vendor instructions.

For all experiments, the PCR assay was run on the ABI 7500 Fast real-time PCR system (Applied Biosciences) using primers and methods to amplify RNase P (RP) (PCR human control), and N1 and N2 SARS-CoV-2 viral targets, as specified in the CDC protocol for SARS-CoV-2 analysis (38, 48). Cycle threshold (*C_T_*) values were recorded for all analyses. All runs had positive, negative, and water blank controls. The criterion for a positive result was *C_T_* values for N1 and N2 of ≤45. The RP control was present to confirm human DNA shedding and also assess degree of inhibition, since it was expected that this marker would be detectable in samples containing human feces. Negative results were interpreted as an indication of PCR inhibition. If positive and negative controls were successful for the run, then a positive could be determined with a negative RP if both viral targets were detected. Samples were deemed negative for SARS-CoV-2 if they exhibited *C_T_* values of >45 for N1 and N2, with a value for RP of ≤45. They were indeterminate when only one viral target was positive.

### Results validation and interpretation.

Patients in the COVID-19 tower on hospital sampling days had undergone PCR-based clinical testing prior to or during admission. Individual patient charts were reviewed to collect dates of initial positive COVID-19 tests and symptom onset, and to compute total occupancy for days when wastewater was collected (Institutional Review Board [IRB] no. 22521).

All occupants of the sampled dormitories were required to undergo midturbinate nasal swab testing (Alinity or m2000; Abbott, Chicago, IL) intermittently throughout the study. Occupants of complexes A and B were tested weekly and biweekly, respectively. Once an occupant tested positive, he or she was isolated for 14 days starting from the test result date and not retested during the study period. The clinical tests were not undertaken as part of this study. The research team did not have access to individual charts for dorm occupants but were instead provided with their testing results in aggregate form. This study was deemed by the IRB to not constitute human subject research.

Sensitivity and specificity were computed via comparison between wastewater-based results and clinical testing results using dorm samples only. Individuals testing positive via clinical testing within the most recent 10-day interval (i.e., contagious period) were considered true positives, and all individuals testing negative via clinical testing within the same interval were considered true negatives. Sensitivity was defined as true positives divided by the sum of true positives and false negatives [TP/(TP + FN)] (49). Specificity was defined as true negatives divided by the sum of false positive and true negatives [TN/(FP + TN)], where a result was considered TN beyond the 10-day period from the initial positive test date and then considered a FP beyond the 10-day contagious period. Sensitivity and specificity were also recalculated using only true positives within the 10-day contagious period (32). Basic statistical calculations were performed using the Microsoft Excel Data Analysis Toolpak (Excel 2019) and R (4.0.2).
